# Fabrication, Potentiometric Characterization, and Application of Screen-Printed RuO_2_ pH Electrodes for Water Quality Testing

**DOI:** 10.3390/s21165399

**Published:** 2021-08-10

**Authors:** Kiranmai Uppuluri, Maryna Lazouskaya, Dorota Szwagierczak, Krzysztof Zaraska, Martti Tamm

**Affiliations:** 1 Łukasiewicz Research Network—Institute of Microelectronics and Photonics, Kraków Division, ul. Zabłocie 39, 30-701 Kraków, Poland; kiranmai.uppuluri@imif.lukasiewicz.gov.pl (K.U.); krzysztof.zaraska@imif.lukasiewicz.gov.pl (K.Z.); 2 Center of Food and Fermentation Technologies, Akadeemia tee 15A, 12618 Tallinn, Estonia; maryna.lazouskaya@tftak.eu (M.L.); martti@tftak.eu (M.T.); 3 Department of Chemistry and Biotechnology, School of Science, Tallinn University of Technology, Ehitajate tee 5, 19086 Tallinn, Estonia

**Keywords:** ruthenium oxide, screen printing, pH electrode, potentiometric sensor, sintering temperature, water pollution monitoring

## Abstract

Screen-printed sensing electrodes attract much attention for water pollution monitoring due to their small size, physical and chemical durability, and low cost. This paper presents the fabrication and broad potentiometric characterization of RuO_2_ pH sensing electrodes deposited by screen printing on alumina substrates and sintered in the 800–900 °C temperature range. All the fabricated electrodes showed close to Nernstian sensitivity, good linearity, fast response, small drift, low hysteresis, and low cross-sensitivity toward various interfering cations and anions. Furthermore, decreasing the sintering temperature led to better adhesion of the RuO_2_ layer and a negligible response to interfering ions. The measurements in real-life samples from different water sources showed that the fabricated electrodes are on par with conventional glass electrodes with a maximum deviation of 0.11 pH units, thus indicating their potential for application in water quality monitoring.

## 1. Introduction

The pollution of rivers and lakes creates a great danger for the environment and human health. Pollution can originate from industrial wastewaters, municipal sewage, and substances used in agriculture (fertilizers, pesticides, and manure). At present, the quality of water is determined against numerous parameters: electrical conductivity, turbidity, dissolved oxygen, toxic inorganic and organic substances, etc. [1]. However, the determination of all the parameters is time-consuming, and sometimes an express test is needed.

A change in pH can be a simple and fast signal of a pollution appearance in water [2,3]. Thus, the availability of cheap, accurate, and stable pH sensors is of high importance. pH sensors, developed in the last decades, vary in detection principles, sensing materials, and fabrication methods [3,4,5,6,7,8,9]. One of the most effective and inexpensive techniques of pH detection is the potentiometric method where the pH of the sample is determined by measuring the potential difference between a pH-sensitive electrode and a reference electrode. The value of the pH is calculated from the Nernst equation [10].

Among the sensing materials investigated for pH detection, metal oxide-based electrodes are well-known for their potential to overcome such drawbacks of glass electrode as fragility (and related risk of contamination with dangerous shattered glass) and poor stability in harsh conditions (strong alkaline or acidic solutions, high pressure, and high temperature) [11,12].

The pH sensing mechanism of metal oxide electrodes is governed by several electrochemical phenomena at the electrode–electrolyte interphase, including adsorption, dissociation, diffusion of ions, hydration, redox processes, electrical double layer formation, and charge transfer [13,14,15,16].

In their fundamental work [13], Fog and Buck analyzed the applicability and pH sensing mechanism of a few metal oxides: PtO_2_, IrO_2_, RuO_2_, OsO_2_, Ta_2_O_5_, and TiO_2_. Among the investigated metal oxides, ruthenium (IV) oxide (RuO_2_) was indicated as the most appropriate material for the pH-sensitive electrodes owing to its mixed electronic-ionic conductivity, relatively low sintering temperature, high sensitivity close to the Nernstian behavior, fast response, low hysteresis, broad pH diapason, resistance to corrosive conditions, chemical and thermal stability, and biocompatibility [6]. Furthermore, among the expensive oxides of the platinum metals group, RuO_2_ is the cheapest one.

Several techniques were used for the deposition of ruthenium oxide-based pH-sensitive electrodes on a substrate. A lot of attention was paid to thin-film methods, including nanostructured film deposition from suspension [17,18], magnetron sputtering [19,20,21,22,23], sol-gel [24,25], the Pechini method [26,27], and electrodeposition [28]. Besides the investigation of pure RuO_2_ layers, numerous studies were devoted to RuO_2_ mixed or doped with other metal oxides (TiO_2_ [26,29], Ta_2_O_5_ [30], SnO_2_ [31], Cu_2_O [18], and La_2_O_3_ [32]), glasses [33,34,35], or carbon nanotubes [20,25].

Among thick-film methods, the most attractive is screen printing [30,35,36,37,38]. This technique is simple, cheap, and flexible in design and manufacturing, and typically provides relatively dense and mechanically strong layers with a thickness ranging from a few to a few tens of micrometers, well-adhering to different substrates. The screen-printing method consists of the deposition of layers of functional materials (metals, glass, and ceramics) on a suitable substrate. The pattern of each layer is fabricated by using a printable thick-film paste and a screen with a stainless steel or polyester mesh that has a predetermined design. A squeegee moves the paste across the screen and forces the material to pass through. After one layer of material has been printed, it undergoes thermal treatment.

The printable material usually consists of conductive, semiconducting, or dielectric particles mixed with binders (e.g., cellulose acetate), solvents (e.g., terpineol), and modifying agents [35,37]. For less demanding applications, cheap polymer-based thick-film pastes cured at low temperatures can be used. However, the best properties of screen printed layers, close to those of bulk functional materials, are attained after complete burnout of organic constituents and subsequent sintering at a proper high temperature (typically 850–900 °C for RuO_2_). To avoid oxidation of metallic components at high temperatures, increase conductivity and/or improve pH sensing performance, noble metal (Pt, Pd, Au) additives are often used in thick-film technology [36]. However, little attention has been paid so far to the influence of the sintering temperature on the properties of the fabricated pH-sensitive electrodes. Moreover, most of the previous research on RuO_2_-based pH electrodes used commercial pastes or mixed oxides and was conducted at a laboratory scale with applications limited to few types of water samples and beverages.

Here, we present the fabrication process and potentiometric investigation of RuO_2_ pH electrodes sintered at three different temperatures: 800, 850, and 900 °C. The areas of investigation include performance characteristics such as sensitivity, response time, drift, hysteresis, and cross-sensitivity with other ions in the solution.

The aim of this work was to verify the applicability of fabricated electrodes for environmental, municipal, and industrial water quality monitoring and investigate their precision. To realize this goal, the pH of real-life samples from different water sources were measured using the RuO_2_ electrodes and compared with the pH values obtained using a glass electrode. Additionally, cross-sensitivity of the fabricated RuO_2_ electrodes to the ionic contaminants that can be found in environmental water due to overfertilization of agricultural lands was evaluated. Another important objective was to show that lowering the sintering temperature, which enables reduction of the environmental and economic impact from the point of view of the firing process, does not negatively impact the performance of the pH electrode.

## 2. Materials and Methods

### 2.1. Preparation of RuO_2_ Paste

Ruthenium oxide paste for screen printing was prepared by mixing anhydrous RuO_2_ powder (density: 6.95 g/cm^3^, Sigma Aldrich, St. Louis, MO, USA) with ethyl cellulose (analytical grade purity) and terpineol (anhydrous, Fluka Analytical, Switzerland) in an agate mortar. Mixing was carried out for 20 min to achieve optimal consistency of the paste.

### 2.2. Fabrication of RuO_2_ Electrodes for Potentiometric Sensors

**Figure 1:**
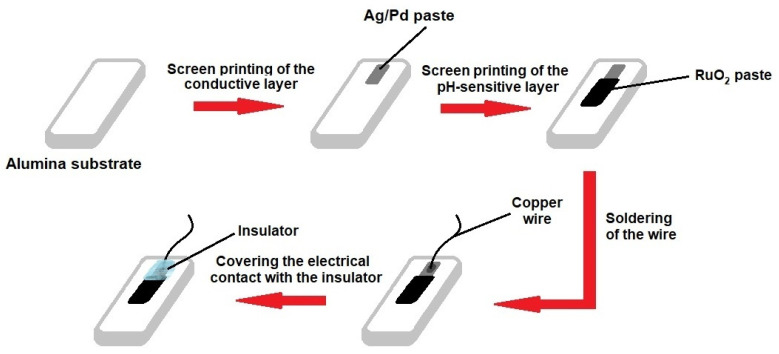
Schematic representation of the fabrication process of RuO_2_ electrodes.

Standard alumina (Al_2_O_3_, 96%) plates were chosen as the substrates for the pH electrodes due to their compatibility with thick films and high tolerance toward various environmental conditions. First, Ag/Pd thick-film paste (9695, Electro-Science Laboratories, King of Prussia, PA, USA) was screen-printed on the substrates, dried at 120 °C for 15 min, and fired at 860 °C for 30 min. Freshly prepared RuO_2_ paste was then screen printed on the substrates in such a way that the RuO_2_ layer slightly overlapped the Ag/Pd conducting layer. After drying at 120 °C for 15 min, electrodes were sintered at 800 °C (RuO_2_-800), 850 °C (RuO_2_-850), or 900 °C (RuO_2_-900) for one hour. Three RuO_2_ electrodes were prepared for each sintering temperature. Next, electrical contact was attached to an open end of the conducting layer by soldering a copper wire. To avoid any contact between the conducting layer and the electrolyte, the electrical contact and the conducting layer were covered with noncorrosive polydimethylsiloxane coating (DOWSIL™ 3140 RTV Coating, Dow Chemical Company, Midland, MI, USA), and the sensitive area was left uncovered. Finally, the silicone resin cover was hardened at room temperature for 48 h. The schematic representation of the various stages of fabrication of RuO_2_ electrodes is presented in Figure 1.

### 2.3. Microstructural Studies

Scanning electron microscopy (SEM) and energy dispersive spectroscopy (EDS) (Nova Nano SEM 200 with EDAX Genesis EDS system, FEI, Hillsboro, OR, USA) were used to examine the microstructure and elemental composition of the fabricated electrodes.

### 2.4. Electrochemical Studies

Electrode potential was measured by standard potentiometric technique. The fabricated RuO_2_ electrodes were used as pH-sensitive electrodes; meanwhile, an ion-selective glass electrode (ISE, Ag|AgCl|KCl, Hydromet, Poland) was used as a reference electrode. To minimize signal loss, electrodes were attached to a unity gain buffer amplifier (LMC6044, Texas Instruments, Dallas, TX, USA) which was further connected to a voltage input module (9205, National Instruments, Austin, TX, USA). LABVIEW software (National Instruments, Austin, TX, USA) was utilized to record all measured data.

#### 2.4.1. pH Measurements

Potentiometric determination of pH relies on selective identification of H^+^ ions present in the investigated solution [39]. The standard potentiometric setup consists of an electrochemical cell and a measuring device (potentiometer, voltmeter, multimeter, etc.). The electrochemical cell consists of a sensing electrode, sensitive to pH change, and a reference electrode (usually silver chloride electrode).

The electrical characteristic of an electrochemical cell is electromotive force (*Emf*). The *Emf* of the cell is determined as the difference in electrode potentials (*E*) of the two half-reactions proceeding at the sensing and reference electrodes. Usually, the reference electrode is grounded, its potential is considered equal to zero, and the *Emf* of the cell is equal to the potential of the sensing electrode.

The half-reaction taking place on the sensing electrode is quantitatively explained by the Nernst equation:

(1) $$E=E^{0}-\frac{R\cdot T}{n\cdot F}\cdot ln\frac{\left[Red\right]}{\left[Ox\right]},$$

where *E*^0^ is standard potential, *V*; *R* is the universal gas constant, 8.314 J/K⋅mol; *T* is temperature, *K*; *n* is the number of the electrodes participating in the redox reaction; *F* is the Faraday constant, 96,485 C/mol; and [Red] and [Ox] are the activities of reduced and oxidized forms of the electrode material, respectively, mol/L. The standard potential is a measure of the individual potential of the reversible electrode (in equilibrium) in the standard state (concentration 1 mol/L, pressure 1 atmosphere and temperature 25 °C).

For the RuO_2_ electrode, the mechanism of pH-sensing can be explained by a simplified equation [11]:

(2) $$Ru^{IV}O_{2}+e+H^{+}↔Ru^{III}O\left(OH\right)$$

The Nernst equation for this process takes the following form:

(3) $$E=E_{Ru^{IV}/Ru^{III}}^{0}-\frac{R\cdot T}{n\cdot F}\cdot ln\frac{\left[Ru^{III}\right]}{[Ru^{IV}]\cdot [H^{+}]},$$

where [*Ru^III^*], [*Ru^IV^*], and [*H*^+^] are activities or *Ru^IV^O*_2_, *Ru^III^O(OH)*, and *H*^+^, respectively, mol/L.

Considering that the values of metal activities approximate 1 in solid-state and substituting the constants, at room temperature (T = 22 °C), Equation (3) takes the following form:

(4) $$E=E_{Ru^{IV}/Ru^{III}}^{0}-0.0583\cdot \mathrm{lg}\left[H^{+}\right]$$

The value of 58.3 mV is called electrode sensitivity or theoretical Nernst response at T = 22 °C. At the given temperature, the sensitivity value should be the same for all the pH-sensitive electrodes when *n* = 1. However, in practice deviation from the theoretical response is observed (see Table S1) [33,34,40].

As a conditioning protocol, all electrodes were immersed in distilled water for 24 h prior to their first measurement in order to hydrate pH-sensitive surfaces.

The sensitivity of the fabricated electrodes was determined by measuring the *Emf* of the electrochemical cell (the potential difference between the reference electrode and the fabricated pH-sensitive electrode) as a function of pH. For that, electrodes were submerged into buffer solutions of pH range from 1 to 14. Buffers were purchased from Chempur (Piekary Śląskie, Poland) and used as received. The pH of the solution was monitored with a combined glass electrode (ELMETRON, Zabrze, Poland).

The *Emf* was recorded for 5 min with data points being collected every 10 s. The *Emf* at the specific pH was determined as the average value of the last 10 data points. Electrode sensitivity, *E*^0^, and linearity of the response were determined by plotting the electrode potential as a function of pH and calculating the equation describing this dependency using the least-squares approach. Electrode sensitivity was calculated as the slope of the linear equation, *E*^0^ was calculated as the potential at pH = 0 by extrapolating the data, and the linearity of the response of the electrode to pH change was calculated as correlation coefficient (R^2^).

#### 2.4.2. Response Time, Drift Rate, and Hysteresis

The response time was determined as the time needed for the electrode potential to reach 90% of the stable value.

To measure the drift of electrode response in time, electrodes were left overnight in distilled water, and the drift rate (in mV/h) was calculated using the slope of the line-of-best-fit approach.

To study the hysteresis, the memory effect of an electrode, the fabricated electrodes were exposed to a series of pH buffers. First, the electrodes were exposed to a pH change from acidic to basic (pH change 1.1 → 4.1 → 7.0 → 10.0 → 13.4), and then, the same pH changes were carried out in the opposite direction. Electrode response was recorded for 3 min after submerging the electrode into a new buffer solution. Electrodes were washed with distilled water and dried with a pressure gun after each measurement.

#### 2.4.3. Cross-Sensitivity

The interference of ions with the performance of the fabricated RuO_2_ electrodes was evaluated by measuring the *Emf* of the cell and determining the sensitivity of the electrodes in the presence of KCl, KNO_3_, NH_4_NO_3_, and (NH_4_)_3_PO_4_. For that, the abovementioned salts were added to buffer solutions to reach the concentration of 0.01 M. Electrodes were immersed into the samples for 5 min, and their *Emf* response was recorded.

### 2.5. Measurements of Real-Life Samples

The fabricated electrodes were used to measure the pH values of different types of water samples: distilled and tap water, mineral water and water from a river and two lakes. Samples were stored at 4 °C prior to any measurement.

Commercially available “Wysowianka” still water was used as mineral water. River water samples were collected from the Vistula River (Kraków, Poland).

**Figure 2:**
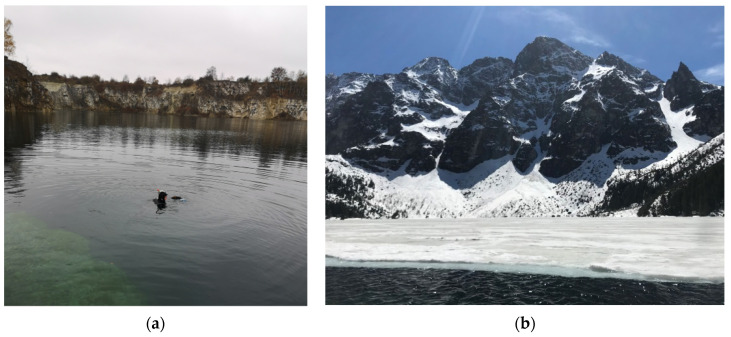
(**a**) Diver collecting samples from Zakrzówek Lake in Kraków, Poland, and (**b**) Lake Morskie Oko in Tatra Mountains, Poland.

Lake water samples were collected from Zakrzówek Lake (Lake Z, Kraków, Poland, Figure 2a) from the surface and the depth of 6 m and the surface of a mountain lake—Lake M (Lake Morskie Oko in Tatra Mountains, Poland, Figure 2b).

## 3. Results and Discussion

### 3.1. Microstructure of RuO_2_ Electrodes

**Figure 3:**
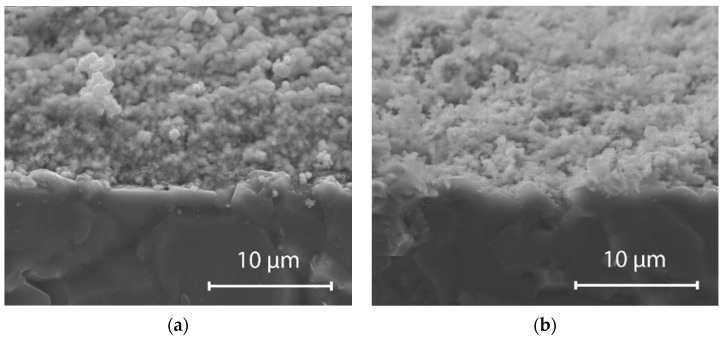
SEM images of the cross-sections of fired electrodes screen printed on alumina substrate: (**a**) RuO_2_-800 and (**b**) RuO_2_-900.

Thermal treatment temperature was previously shown to affect the properties of metal oxide solid-state electrodes due to change in relative density and/or crystallinity with temperature. The sensing layer morphology significantly impacts its sensing performance [17,21,41,42]. For the fabricated RuO_2_ electrodes, there was only a slight change in surface morphology and microstructure of the layers sintered at different temperatures in the range of 800–900 °C. The EDS analysis confirmed the presence of both ruthenium and oxygen in the fabricated pH-sensitive layers. SEM images of fractured cross-sections of the electrodes screen printed on Al_2_O_3_ substrates (Figure 3) indicated that both the RuO_2_-800 and the RuO_2_-900 electrodes were characterized by small porosity and uniform, fine-grained microstructure with grain sizes of 0.5–2 µm. However, for RuO_2_-800 electrodes (Figure 3a), improved adhesion to the substrate was observed, probably due to their lower porosity related to densification proceeding with a higher contribution of the amorphous phase at grain boundaries. Higher content of pores in the sensing layer can entail scattering of the charge carriers, leading to reduced carrier mobility and decreased sensitivity [41].

### 3.2. Sensitivity of the Fabricated Electrodes

**Figure 4:**
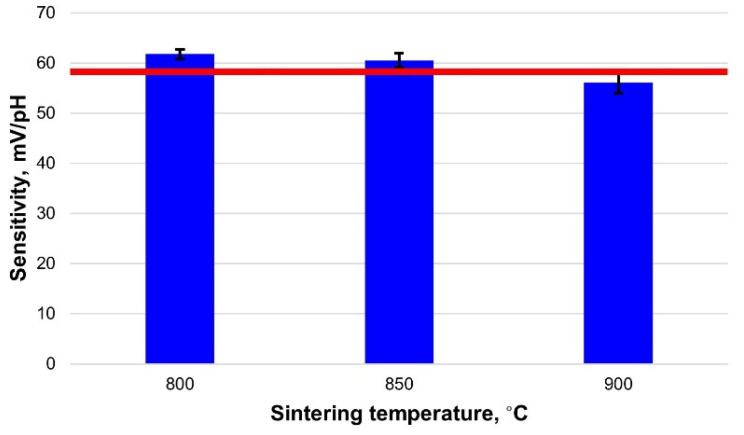
Comparison of the sensitivity of the fabricated electrodes sintered at different temperatures with the theoretical Nernstian response (red line).

Sensitivity is the key characteristic of an electrode that allows determining if the electrode is working properly. The pH sensitivity of the fabricated RuO_2_ electrode was determined by exposing the electrodes to buffer solutions of different pH and calculating the slope of the Nernst equation for the electrodes based on measured *Emf*. The results are presented in Figure 4 and Table 1. It can be seen that the sensitivity is close to the theoretical response with good linearity (R^2^ = 0.994 − 0.996) for all the electrodes. Furthermore, the sensitivity values are slightly decreasing with an increase in sintering temperature. Therefore, decreasing the sintering temperature of the RuO_2_ pH electrodes does not negatively impact their sensitivity and can be implemented to reduce the power consumption that is needed to achieve higher firing temperatures in the furnace.

The sensitivity of the pH electrodes under investigation became stable almost immediately. Moreover, the response is faster than that of the RuO_2_ electrodes fabricated from the commercial paste containing a glass addition [43].

### 3.3. Long-Term Stability and Repeatability

**Figure 5:**
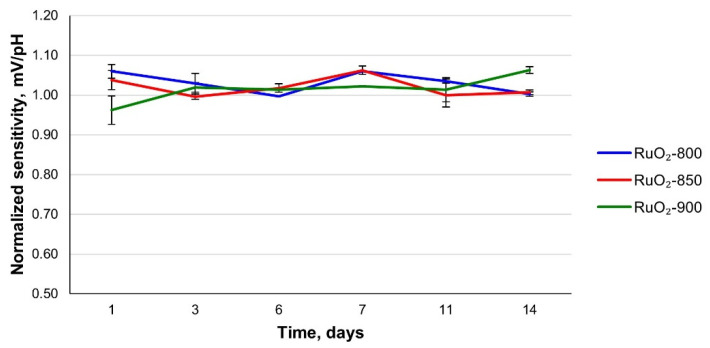
Sensitivity of the RuO_2_-800, RuO_2_-850, and RuO_2_-900 electrodes as a function of time.

It was previously shown that solid-state electrodes might require some time to reach stable sensitivity values [17,29]. However, no significant change in sensitivity for all the fabricated electrodes was noticed over two weeks of usage (Figure 5, Table S2). The daily variation in temperature was taken into account, and the observed sensitivity values were normalized according to expected theoretical sensitivity at a given temperature. The sensitivity values of RuO_2_-800, RuO_2_-850, and RuO_2_-900 electrodes remained close to the theoretical value and did not significantly change over time. Furthermore, the decrease in sensitivity with sintering temperature, observed during the first usage of the electrodes, did not occur during further measurements.

The porosity and surface oxidation states of the RuO_2_ layer can be impacted due to the ageing of an electrode [17]. Nevertheless, in our study, ageing did not impact the pH sensing properties of the electrodes even though they were only conditioned once before the first measurement. The pH sensitivity of RuO_2_-850 and RuO_2_-900 electrodes fabricated six months earlier and stored in the air was only reduced by 2.7 and 6.5 mV/pH, respectively. For general water quality monitoring, the pH is not expected to change very rapidly and frequently. However, the fabricated electrodes were repeatedly subjected to fast changes in pH range 1–13.4. The stability in sensitivity over days thereby exhibits the endurance of RuO_2_ to multiple measurements in electrolytes with varying pH across a wide range. Therefore, fabricated electrodes exhibit not only long-term stability in operation but also have a long shelf life. Moreover, the loss in sensitivity over 6 months was lower at a lower sintering temperature.

### 3.4. Drift and Response Time

For continuous measurements that can last for hours, an important characteristic of an electrode is the drift rate. Drift rate is used to evaluate if the readings of an electrode maintain the same over a long period of observation. The values of the drift rate for the electrodes are presented in Table 1. These values remained small and did not exceed 1 mV/h.

Both the potential drift of an electrode and the response time are closely related to the mechanisms that govern them. Based on the material composition, drift of the *Emf* during pH measurement in RuO_2_ electrodes can be caused due to slow hydration of the pH-sensitive layer or slow H^+^ ions diffusion [17]. Other critical aspects of the RuO_2_ electrode that determine the drift of potential are homogeneity, porosity and thickness of the pH-sensitive layer, and composition and structure of the pH-sensitive material [17]. For a given temperature and electrode composition, the drift rate is expected to be small as it does not significantly depend on the interface between the pH-sensitive surface and the electrolyte, while pores at grain boundaries trapping H_2_ govern the H^+^ ions transport through this layer [17]. Therefore, the low drift rate related to fast surface hydration and H^+^ diffusion experienced by the fabricated RuO_2_ electrodes could be attributed to their single-phase composition without additives and the uniform microstructure.

Response time is the characteristic that tells how long one needs to wait before the electrode reaches stable potential readings. Response time plays an important role when a fast change in pH must be detected. All the fabricated electrodes showed a response time of 2–36 s (Table 1).

The disadvantage of pure RuO_2_ could be lesser adhesion to the substrate [17]. To overcome it, glass can be added to the screen-printing paste [33,35]. However, the addition of glass has been reported to increase the response time due to reduced porosity of the printed layer and diffusion rate of protons [6]. The choice of the best RuO_2_-based composition is therefore strongly dependent on the requirements of the user and the area of the sensor application. In our research, we have similarly experienced that the layer made of commercial RuO_2_ paste containing glass particles adheres better to the alumina substrate than a layer of pure RuO_2_. However, the slightly weaker mechanical strength and integrity of the latter do not impact its sensitivity to pH as shown in Section 3.2.

The response at the pH < 7.0 is almost instantaneous (2 s). However, as the pH increases, the response time also increases, and at pH 13, the maximum response time of 52 s is observed. The negative effect of pH increase on the response time can be attributed to the low concentration of H^+^ ions at higher pH values [6]. Furthermore, compared to the OH^−^ ions that are bigger and diffuse more slowly, H^+^ ions are small, which makes their diffusion in the RuO_2_ layer faster and therefore facilitates relevant ion exchange [6].

### 3.5. Hysteresis

**Figure 6:**
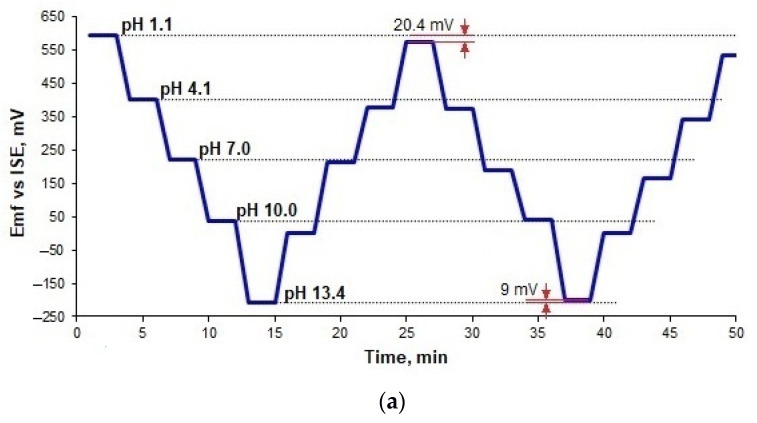

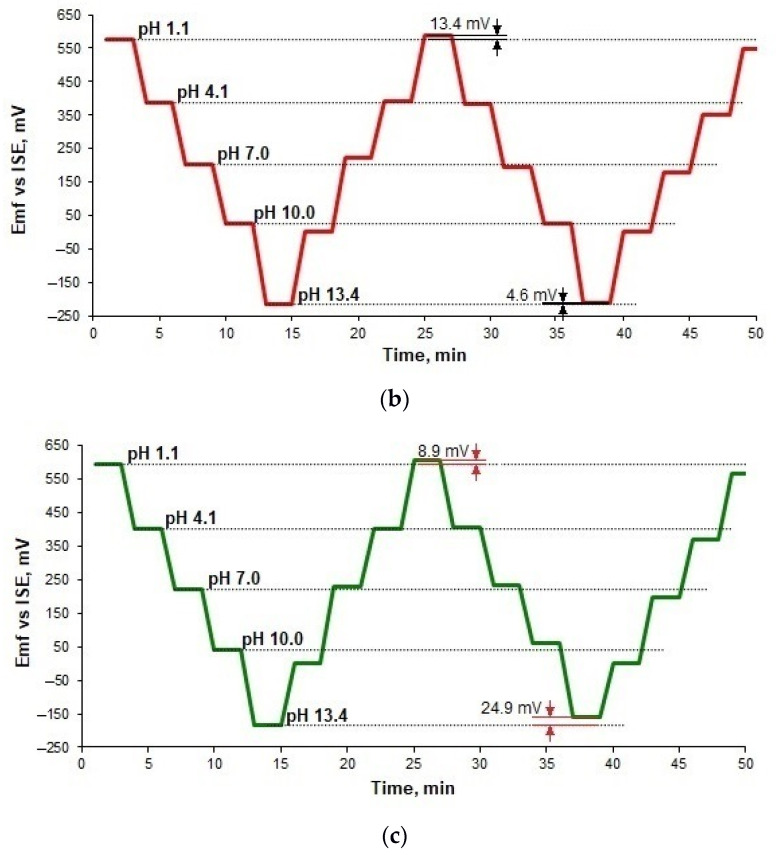
Hysteresis of the RuO_2_-800 (**a**), RuO_2_-850 (**b**), and RuO_2_-900 (**c**) electrodes for the pH 1.1→4.1→7→10→13.4→10→7→4.1→1.1→4.1→7→10→13.4→10→7→4.1→1.1 loop.

Another important characteristic of an electrode is hysteresis. Differences in the *Emf* values at the same pH occur when the measurement is repeated multiple times. The analysis of this phenomenon, also known as the memory effect, allows for estimating if the previous measurement affects the consequent. To evaluate the hysteresis, the fabricated electrodes were exposed to a series of pH buffer changes. The RuO_2_ electrodes exhibited a small hysteresis effect (Figure 6). All the fabricated electrodes showed hysteresis values not exceeding 21 mV when the pH changing started from the acidic region (1.1 → 13.4 → 1.1). For the opposite direction of pH change (13.4 → 1.1 → 13.4), the hysteresis values did not exceed 25 mV. These results show that all electrodes have a proper response to pH changes. The lowest hysteresis was observed for the RuO_2_-850 electrodes.

The pH measurement loop and crystalline properties of the pH-sensitive material are two factors that mainly influence the hysteresis effect in metal oxide-based pH electrodes [6]. The hysteresis effect in this study was higher for all electrodes when pH change started from the basic to acidic region (13.4 → 1.1 → 13.4) because OH^−^ ions H^+^ ions diffuse slower than H^+^ ions in the RuO_2_ layer. This finding is in agreement with previous studies [6,20,23]. However, it can be seen that similar to the response time, the hysteresis effect was also lower for higher sintering temperature, which may be attributed to higher porosity.

### 3.6. Cross-Sensitivity

One of the main limitations of the usage of metal oxide pH electrodes is the sensitivity of the electrode to interfering ions. The interferences caused by various ions were investigated by measuring the sensitivity in the presence of K^+^ and NH_4_^+^ cations and Cl^−^, NO_3_^−^, and PO_4_^3−^ anions. The observed pH sensitivities in the presence of the interfering ions for all three types of electrodes are listed in Table 2. Furthermore, Table S1 presents the comparison of electrode properties and sensitivity characteristics of RuO_2_-based pH sensors reported previously by other authors with those obtained in this work.

**Figure 7:**
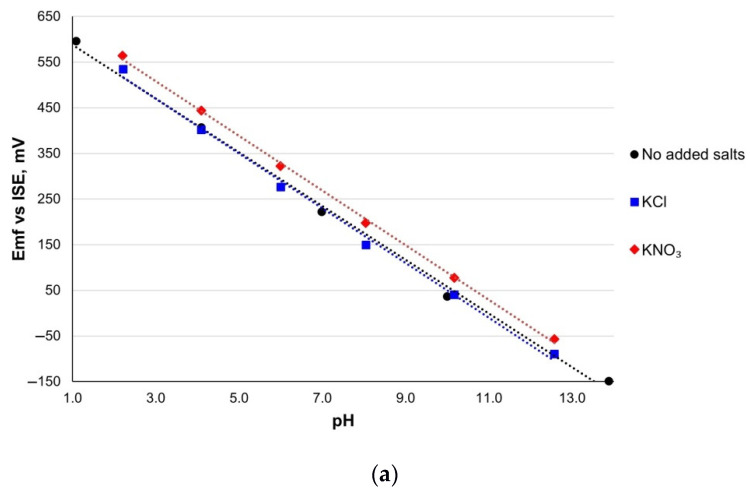

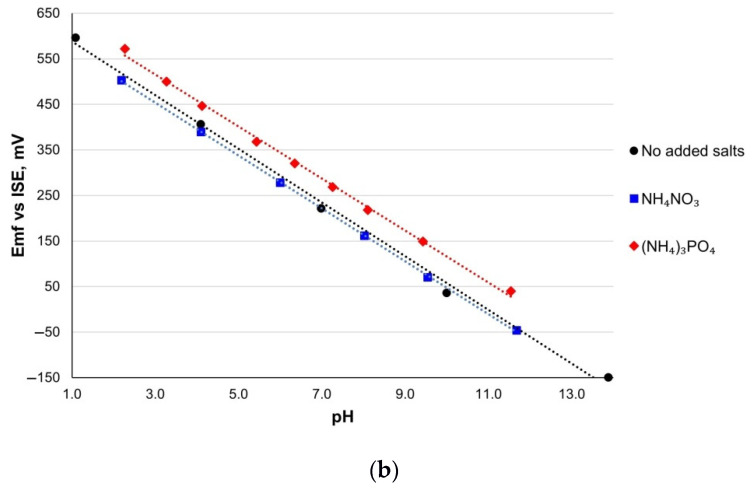
The electrochemical response of the RuO_2_-800 electrodes to the pH change in the presence of (**a**) potassium salts: (**a**) KCl and KNO_3_ and (**b**) ammonia salts: NH_4_NO_3_ and (NH_4_)_3_PO_4_ in comparison with the response without added salts.

The *Emf* versus pH plots for the RuO_2_-800 electrode in the presence of interfering ions are presented in Figure 7. For RuO_2_-850 and RuO_2_-900 electrodes, similar behavior was observed. All the fabricated electrodes exhibited close to the Nernstian response and did not show any significant deviation from the theoretical sensitivity in distilled water when no salt was added (Table 2).

In general, the effect of the interfering salts was small, especially for RuO_2_-800 sensing electrodes fired at the optimal temperature of 800 °C. However, in the case of RuO_2_-850 and RuO_2_-900 electrodes, a decrease in sensitivity was observed. The maximum impact observed was a reduction of 5.1 mV/pH for RuO_2_-850 in the presence of 0.01 mol/L of KCl. K^+^ ion is smaller in size as compared to NH_4_^+^, and therefore, it has higher mobility. Due to its faster diffusion into the double layer at the sensing surface of the RuO_2_ electrode, it influences the sensitivity of the electrode more than the bigger and slower NH_4_^+^ cation. The most distinct deviation in the E^0^ potential observed for (NH_4_)_3_PO_4_ (for RuO_2_-800 and RuO_2_-900 electrodes) implies that the phosphate changes the contribution of Ru^4+^ and Ru^3+^ ions governing the redox reaction in the sensing layer. Such shifts in the E^0^ values were attributed by Lonsdale et al. [21] to the changes in the Ru^4+^/Ru^3+^ ratio caused by oxidizing/reducing agents.

The lack of interference from various ions in the solution may be due to the undoped composition of the sensing layer based on pure RuO_2_ used to fabricate the sensors. In the study by Labrador et al. [38], where commercial RuO_2_ paste was used, interference from halides, carbonates, and sulfates were attributed to the presence of lead (II) oxide (PbO), which causes the formation of insoluble products with the anions. Commercial RuO_2_ paste contains lead borosilicates because it aids in modifying material properties such as temperature coefficient of expansion of the glass phase [44]. Pocrifka et al. [26] reported a pH electrode developed by mixing RuO_2_ with 30 mol.% TiO_2_ that was insensitive toward cations (Li^+^, Na^+^, and Ca^2+^) without a significant improvement in the pH sensitivity when compared to the undoped RuO_2_ electrode.

In this study, it was stated that a pure RuO_2_ sensing electrode is capable of being uninfluenced by various interfering ions without the addition of lead borosilicate-based glass phase or TiO_2_. This indicates that the addition of PbO to RuO_2_ only assists in improving the thick-film properties of the pH electrode, such as sheet resistivity, adhesion, low temperature coefficient of resistance, and low noise indices [44]. Similarly, the advantage of adding TiO_2_ is less related to improvement in sensitivity and selectivity, and it has more to do with lowering the cost [26] and avoiding corrosion by oxidation of RuO_2_ to RuO_4_ or RuO_4_^2−^ [45]. Therefore, the additives to pure RuO_2_ may have an adverse, favorable, or neutral impact on the cross-sensitivity of the pH electrode depending on the properties of the additive, and the decision of their use rests upon the requirements and priorities of the researchers and manufacturers.

All the compounds used in the cross-sensitivity experiment performed in this study are also agricultural fertilizers used for plant growth, and the combined presence of nitrogen, phosphorus, and potassium in the composition gives such fertilizers the common name N-P-K fertilizers. Albeit their great contribution to tackling the ever-growing global demand for food, N-P-K fertilizers are also notorious for nutrient pollution through groundwater contamination and surface run-off from farms to in-land waters. In order to be absorbed by the plant’s root hair cells, fertilizers must be soluble in water, and the water solubility of KNO_3_, KCl, and NH_4_NO_3_ is 100%, whereas for (NH_4_)_3_PO_4_, it is 35% [46]. Therefore, the low cross-sensitivity of fabricated RuO_2_ pH electrodes makes them suitable for measurement in water that has been contaminated by fertilizers and is rich in nutrients. Applicability of such pH sensing without interference from ions such as K^+^, NO_3_^-^, NH^4+^, and PO_4_^3−^ ranges from water quality monitoring in agriculture to municipal and industrial wastewater treatment.

### 3.7. Performance of pH Electrodes in Real-Life Water Samples

The fabricated electrodes were tested in several water samples to evaluate their applicability to real-life measurements. The results obtained for distilled, mineral, tap, river, and lake water are presented in Table 3. The pH values measured with the fabricated electrodes were similar to those measured with a conventional glass electrode with the maximum discrepancy of 0.11 pH units observed for Lake M water samples. Furthermore, all the fabricated electrodes showed good uniformity of the measured pH value, with the standard deviation not exceeding 0.13 pH units.

The highest difference between the measurement by glass electrode and the fabricated RuO_2_ electrodes was only 0.11 units for surface water of Lake Z and Lake M measured by RuO_2_-900 electrode. The RuO_2_-850 electrode exhibited the closest to the glass electrode pH value and a very small variation (±0.01 pH units) in parallel readings for all water samples except Lake M water.

The industrially manufactured and commercially available mineral water tested in this study contains cations such as Na^+^, Ca^+^, Mg^+^, and K^+^, and anions such as HCO_3_^−^, SO_4_^2−^, Cl^−^, and F^−^. In 2017, an analysis of municipal tap water in Krakow, Poland, revealed the presence of calcium, magnesium, and nitrates in it [47]. The quality of water in the Vistula River is quite impacted by urbanization, and a high content of contaminants such as NH_4_^+^, K^+^, Ca^2+^, Mg^2+^, Zn^2+^, Mn^2+^, Fe^2+^, Fe^3+^, etc., can be found in it [48]. Until 1991, Lake Z was an upper Jurassic limestone quarry, and a 2003 study by Galas [49] reported high conductivity and chloride content of water due to infiltration from the nearby Vistula River. Lake M was reported to have a cadmium concentration of about 0.13 mg/dm^3^ due to anthropogenic activities [50].

The obtained results of the measurements of the water samples highlight the effectiveness of the fabricated RuO_2_ pH electrodes in the presence of various chemical species and elements. Therefore, reported electrodes are capable of functioning accurately in environmental, industrial, and municipal water quality monitoring.

## 4. Conclusions

Thick-film pH-sensitive electrodes based on pure RuO_2_ without the addition of glass or other metal oxides were successfully prepared by screen printing and subsequent sintering at different temperatures. Excellent sensing characteristics were attained owing to the single-phase composition of the electrodes, their high conductivity, and good electrochemical and catalytic properties of RuO_2_. The advantageous properties comprised the Nernstian behavior in a broad pH range of 1–13 with the sensitivity of 56.1–61.8 mV/pH, fast response of 2 s for pH ≤ 7, low hysteresis, good long-term stability, and high resistance to cross-sensitivity from various interfering cations and anions. The analysis of the influence of sintering temperature of RuO_2_ electrodes on the performance of potentiometric pH electrodes showed small differences for the samples sintered at 800, 850, and 900 °C. However, a sintering temperature of 800 °C should be preferred not only from the economic and ecological point of view but also for the better adhesion of the RuO_2_ layer to the alumina substrate and its lowest cross-sensitivity. Furthermore, the excellent applicability of the fabricated electrodes for the pH measurements of real-life water samples (tap, mineral, river, and lake water) was proved in this work. The water samples used in the study exposed the RuO_2_ pH-sensitive layers to a variety of chemical elements, and the precise pH measurement in such conditions showed their ability to function correctly in different environments. In most water samples, the electrodes sintered at 850 °C showed the best precision and repeatability. The developed screen-printed potentiometric electrodes seem to be promising candidates for online monitoring systems of water quality due to their excellent pH sensing performance and potential for miniaturization and wireless measurement data transfer.

## Figures and Tables

**Table 1:** Potentiometric characteristics of the fabricated electrodes.

Electrode Type	Sensitivity, mV/pH		*E*^0^, mV	R^2^	Response Time, s		Drift Rate, mV/h
	Theoretical	Observed			pH ≤ 7	pH > 7	
RuO_2_-800	58.9	61.8 ± 1.0	681.9 ± 5.0	0.996	2 ± 0	36 ± 1	0.1–0.2
RuO_2_-850		60.5 ± 1.4	664.2 ± 11.3	0.994	2 ± 0	34 ± 0	0.1–0.9
RuO_2_-900		56.1 ± 2.1	624.0 ± 27.9	0.996	2 ± 0	26 ± 2	0.1–0.4

**Table 2:** Sensitivity characteristics of the electrodes in presence of interfering salts.

	Sensitivity, mV/pH	*E*^0^, mV	R^2^
RuO_2_-800			
No added salt	59.1 ± 0.2	647.0 ± 0.1	0.982
KCl	58.9 ± 1.1	630.3 ± 18.9	0.992
KNO_3_	59.7 ± 0.2	684.3 ± 3.8	0.999
NH_4_NO_3_	58.5 ± 0.6	633.9 ± 6.5	1.000
(NH_4_)_3_PO_4_	58.0 ± 2.3	686.15 ± 0.0	0.998
RuO_2_-850			
No added salt	59.0 ± 0.4	642.4 ± 0.7	0.998
KCl	55.8 ± 0.2	585.3 ± 41.7	0.996
KNO_3_	54.4 ± 2.2	550.8 ± 100.1	0.997
NH_4_NO_3_	59.0 ± 0.8	642.0 ± 0.2	1.000
(NH_4_)_3_PO_4_	57.0 ± 1.4	654.3 ± 16.1	0.995
RuO_2_-900			
No added salt	61.5 ± 1.1	645.6 ± 17.0	0.998
KCl	56.4 ± 0.6	642.3 ± 14.5	0.999
KNO_3_	58.0 ± 0.7	674.9 ± 14.2	0.999
NH_4_NO_3_	57.9 ± 0.3	629.4 ± 1.3	0.999
(NH_4_)_3_PO_4_	59.9 ± 1.0	695.2 ± 13.1	0.996

**Table 3:** pH values of water samples measured with a glass electrode and the fabricated RuO_2_ electrodes.

Water Sample	pH Values Measured with			
	Glass Electrode	RuO_2_-800	RuO_2_-850	RuO_2_-900
Distilled water	7.30	7.29 ± 0.04	7.29 ± 0.01	7.27 ± 0.00
Mineral water	5.74	5.75 ± 0.01	5.74 ± 0.01	5.75 ± 0.00
Tap water	8.32	8.35 ± 0.05	8.39 ± 0.01	8.33 ± 0.07
River water	8.05	8.12 ± 0.01	8.11 ± 0.00	8.12 ± 0.00
Lake Z water (surface)	8.04	7.97 ± 0.11	8.04 ± 0.01	7.93 ± 0.02
Lake Z water (deep)	8.12	8.06 ± 0.07	8.13 ± 0.01	8.08 ± 0.11
Lake M water (surface)	6.75	6.84 ± 0.08	6.83 ± 0.11	6.86 ± 0.13

## Data Availability

Data are contained within the article or the Supplementary Materials. The LabView block diagram is available upon request from the authors.

## Supplementary Materials

The following are available online at https://www.mdpi.com/article/10.3390/s21165399/s1: Table S1. Summary of RuO_2_-based pH electrode characteristics reported previously and in this work, Table S2. Change of the sensitivity of the fabricated electrodes with time.
