# Supplementary material for: Fabrication, Potentiometric Characterization, and Application of Screen-Printed RuO2 pH Electrodes for Water Quality Testing
Source: Sensors (Basel). 2021 Aug 10;21(16):5399. doi: 10.3390/s21165399 (PMC8401146; doi:10.3390/s21165399)
Supplement: Supplementary file 1 [file sensors-21-05399-s001.zip › sensors-1323077-supplementary.pdf]

Supplementary Material

Table S1. Summary of RuO_2 based pH electrode characteristics reported previously and in this work

Deposition method	Electrode sensitive material	Substrate	Precursor	Slope, mV/pH	E^0, mV	R^2	pH range	Response time, s	T, ^0C	Hysteresis, mV	Drift, mV/h	Reference electrode	Ref.
ED [1]	RuO_2	Au wire	K_2RuO_4	59.3	521	N/A	2.0 - 12.0	N/A	N/A	N/A	N/A	NHE [2]	[1]
		Au disk	$RuCl_3{\cdot}xH_2O$	60.5	N/A	0.999	2.0 – 11.0	7	N/A	N/A	N/A	Ag|AgCl|KCl	[2]
		Pt	RuO_2	56.2	596	0.999	4.0 - 10.0	N/A	37.5	N/A	N/A		[3]
		Pt-Ti	RuO_2	59.3	609	0.999	4.0 - 10.0	N/A	37.5	N/A	N/A		[3]
Screen-printing	RuO_2	Polyester foil	RuO_2-graphite	51.2	606	N/A	2.0 – 10.0	N/A	N/A	N/A	N/A	SCE [3]	[4]
		Al_2O_3	RuO_2	58.4	578	0.995	2.0 – 12.0	14	22	N/A	0.5	Ag|AgCl|KCl	[5]
		Al_2O_3	$RuO_2{\cdot}xH_2O$	52.1	651	N/A	2.0 – 10.0	15	21 – 24	N/A	N/A		[6]
		**Al_2O_3**	**RuO_2**	**61.8**	**682**	**0.996**	**1.0 – 13.0**	**14**	**22**	**4.6 – 24.9**	**0.15**		**This study**
		Al_2O_3	RuO_2-glass [4]	56.0	N/A	N/A	2.0 – 12.0	120	N/A	N/A	N/A	Screen printed Ag/AgCl	[7]
	RuO_2-Ta_2O_5	Al_2O_3	RuO_2, Ta_2O_5, glass powder	68.2	N/A	0.987	2.0 – 12.0	15	N/A	10	N/A	Screen printed Ag/AgCl	[7]
	RuO_2-Ta_2O_5	Al_2O_3	RuO_2, Ta_2O_5	56	N/A	N/A	2.0 – 12.0	15	N/A	N/A	N/A	Ag|AgCl|KCl	[8]
	RuO_2-SnO_2	Al_2O_3	RuO_2, SnO_2	56.5	631	0.998	2.0 – 12.0	9	N/A	7	N/A		[9]
	RuO_2-Cu_2O	Al_2O_3	RuO_2, Cu_2O	47.4	N/A	N/A	2.0 – 13.0	N/A	21	N/A	N/A		[10]
	RuO_2-La_2O	Al_2O_3	RuO_2, La_2O_3	49.3	N/A	N/A	2.0 – 12.0	N/A	19	N/A	N/A		[11]
	RuO_2-TiO_2	Al_2O_3	RuO_2, TiO_2	56.6	630	0.999	2.0 – 11.0	15	N/A	5	N/A		[12]
	RuO_2-Pt	Al_2O_3-Pt	RuO_2	58.0	640	N/A	2.0 – 13.0	1-2	23	~ 0	1.5		[13]
	RuO_2-Nafion	Al_2O_3	RuO_2	58.3	656	0.999	2.0 – 12.0	42	22	N/A	0.4		[5]
	RuO_2-Nafion	Al_2O_3	RuO_2-glass [4]	58.6	684	0.995	3.0 – 11.0	N/A	N/A	11.5 – 26	N/A		[14]

Deposition method	Electrode sensitive material	Substrate	Precursor	Slope, mV/pH	E^0, mV	R^2	pH range	Response time, s	T, ^{0}C	Hysteresis, mV	Drift, mV/h	Reference electrode	Ref.
Sol-gel	RuO_2-CN	Au, Co, steel	RuO_2, CNT	63.1	647	1.000	2.0 – 12.0	50	N/A	N/A	N/A	SCE	[15]
Pechini	RuO_2-TiO_2	Ti	$RuCl_3$, Ti	56.0	N/A	0.998	2.0 – 12.0	N/A	25	N/A	N/A		[16]
RFMS [5]	RuO_2	Pt wire	RuO_2-glass [6]	60	913	N/A	2.0 – 12.0	90	25	30	3	NHE	[17]
		OMC [7]	RuO_2	57.8	598	0.999	2.0 – 12.0	180	22	3.14	19.0	Ag|AgCl|KCl	[18]
		OMC	RuO_2	58.4	670	0.999	4.0 – 10.0	30	22	1.13	5.0		[19]
		Carbon	RuO_2	59.2	800	1.000	4.0 – 10.0	25	22	5.44	20.5		[19]
		Pt	RuO_2	58.6	925	0.999	4.0 – 10.0	20	22	6.45	23.4		[19]
		Si	Ru	55.6	N/A	N/A	1.0 – 13.0	<1	N/A	4.36	0.38		[20]
		Si	Ru	51.7	N/A	0.978	2.0 – 10.0	N/A	N/A	N/A	N/A	SCE	[21]
		Si	RuO_2	55.8	N/A	0.998	2.0 – 10.0	N/A	N/A	N/A	N/A		[21]
		Si	Ru	56.0	N/A	N/A	1.0 - 12.0	N/A	N/A	N/A	N/A	Ag|AgCl|KCl	[22]
		Al_2O_3	RuO_2	73.8	N/A	0.998	4.0 - 10.0	3	22	~ 5	N/A		[23]
		Al_2O_3	RuO_2	58.8	N/A	0.999	2.0 – 12.0	30	22	1.3	2.9	RuO_2 [8]	[24]
	RuO_2-Ta_2O_5-Nafion	Al_2O_3	RuO_2	55.3	288	1.000	2.0 – 12.0	136 (pH 6)	22	0.7	7.2	RuO_2 [8]	[24]
	RuO_2-CNT [9]	Ta	Ru, CNT	55.5	643	1.000	2.0 – 12.0	40	25	10.2	~ 3	Ag|AgCl|KCl	[25]

[1] ED – Electrodeposition
[2] NHE – Normal Hydrogen Electrode
[3] SCE – Saturated calomel electrode
[4] RuO_2-glass paste with the resistivity of 10 kΩ/sq (3914, Electro-Science Laboratories, USA)
[5] RFMS – Radio-Frequency Magnetron Sputtering
[6] Submicron particles of RuO_2 and high lead silicate, or borosilicate, glass (typically containing 63-90% PbO, 10-25% SiO_2 and 0-25% B_20_3, by weight) dispersed in a viscous organic liquid This study
[7] OMC - Ordered Mesoporous Carbon
[8] RuO_2, acrylic well filled with PVB-SiO_2 junction material
[9] CNT – Carbon Nanotubes

Table S2. Change of the sensitivity of the fabricated electrodes with time

Time, days	Temperature, °C	Theoretical sensitivity, mV/pH	Measured sensitivity, mV/pH		
			RuO_2-800	RuO_2-850	RuO_2-900
1	21	58.3	61.8 ± 1.0	60.5 ± 1.4	56.1 ± 2.1
3	21	58.3	60.0 ± 1.5	58.1 ± 0.4	59.4 ± 0.7
6	22	58.5	58.3 ± 0.0	59.5 ± 0.6	59.3 ± 0.0
7	22	58.5	62.0 ± 0.1	62.1 ± 0.6	59.8 ± 0.1
11	22	58.5	60.6 ± 0.3	58.5 ± 1.8	59.3 ± 1.8
14	24	58.9	58.7 ± 0.3	58.9 ± 0.3	62.2 ± 0.5

References

1. Pasztor, K.; Sekiguchi, A.; Shimo, N.; Kitamura, N.; Masuhara, H. Electrochemically-deposited RuO_2 films as pH sensors. *Sens. Actuators B Chem.* **1993**, *14*, 561–562, doi:10.1016/0925-4005(93)85091-n.
2. Shim, J.H.; Kang, M.; Lee, Y.; Lee, C. A nanoporous ruthenium oxide framework for amperometric sensing of glucose and potentiometric sensing of pH. *Microchim. Acta* **2012**, *177*, 211–219, doi:10.1007/s00604-012-0774-9.
3. Mingels, R.; Kalsi, S.; Cheong, Y.; Morgan, H. Iridium and Ruthenium oxide miniature pH sensors: Long-term performance. *Sens. Actuators B Chem.* **2019**, *297*, 126779, doi:10.1016/j.snb.2019.126779.
4. Koncki, R.; Mascini, M. Screen-printed ruthenium dioxide electrodes for pH measurements. *Anal. Chim. Acta* **1997**, *351*, 143–149, doi:10.1016/s0003-2670(97)00367-x.
5. Uppuluri, K.; Lazouskaya, M.; Szwagierczak, D.; Zaraska, K. Influence of temperature on the performance of Nafion coated RuO_2 based pH electrodes. In Proceedings of the 2021 IEEE International Conference on Flexible and Printable Sensors and Systems (FLEPS), Manchester, UK, 20–23 June 2021; pp. 1–4.
6. Mihell, J.; Atkinson, J. Planar thick-film pH electrodes based on ruthenium dioxide hydrate. *Sens. Actuators B Chem.* **1998**, *48*, 505–511, doi:10.1016/s0925-4005(98)00090-2.
7. Manjakkal, L.; Synkiewicz, B.; Zaraska, K.; Cvejin, K.; Kulawik, J.; Szwagierczak, D. Development and characterization of miniaturized LTCC pH sensors with RuO_2 based sensing electrodes. *Sens. Actuators B Chem.* **2016**, *223*, 641–649, doi:10.1016/j.snb.2015.09.135.
8. Manjakkal, L.; Cvejin, K.; Kulawik, J.; Zaraska, K.; Socha, R.P.; Szwagierczak, D. X-ray photoelectron spectroscopic and electrochemical impedance spectroscopic analysis of RuO_2-Ta_2O_5 thick film pH sensors. *Anal. Chim. Acta* **2016**, *931*, 47–56, doi:10.1016/j.aca.2016.05.012.
9. Manjakkal, L.; Cvejin, K.; Kulawik, J.; Zaraska, K.; Szwagierczak, D.; Stojanovic, G. Sensing mechanism of RuO_2–SnO_2 thick film pH sensors studied by potentiometric method and electrochemical impedance spectroscopy. *J. Electroanal. Chem.* **2015**, *759*, 82–90, doi:10.1016/j.jelechem.2015.10.036.
10. Zhuiykov, S.; Kats, E.; Marney, D.; Kalantar-Zadeh, K. Improved antifouling resistance of electrochemical water quality sensors based on Cu_2O-doped RuO_2 sensing electrode. *Prog. Org. Coat.* **2011**, *70*, 67–73, doi:10.1016/j.porgcoat.2010.10.003.
11. Zhuiykov, S.; Kats, E.; Marney, D.; Kalantar-Zadeh, K. Improved antifouling resistance of electrochemical water quality sensors based on Cu_2O-doped RuO_2 sensing electrode. *Prog. Org. Coat.* **2011**, *70*, 67–73, doi:10.1016/j.porgcoat.2010.10.003.
12. Manjakkal, L.; Cvejin, K.; Kulawik, J.; Zaraska, K.; Szwagierczak, D.; Socha, R.P. Fabrication of thick film sensitive RuO_2-TiO_2 and Ag/AgCl/KCl reference electrodes and their application for pH measurements. *Sens. Actuators B Chem.* **2014**, *204*, 57–67, doi:10.1016/j.snb.2014.07.067.
13. Zhuiykov, S. Morphology of Pt-doped nanofabricated RuO_2 sensing electrodes and their properties in water quality monitoring sensors. *Sens. Actuators B Chem.* **2009**, *136*, 248–256, doi:10.1016/j.snb.2008.10.030.
14. Lazouskaya, M.; Tamm, M.; Scheler, O.; Uppuluri, K.; Zaraska, K. Nafion as a protective membrane for screen-printed pH-sensitive ruthenium oxide electrodes. In Proceedings of the 2020 17th Biennial Baltic Electronics Conference (BEC), Tallinn, Estonia, 6–8 October 2020; pp. 1–4.
15. Kahram, M.; Asnavandi, M.; Dolati, A. Synthesis and electrochemical characterization of sol–gel-derived RuO_2/carbon nanotube composites. *J. Solid State Electrochem.* **2013**, *18*, 993–1003, doi:10.1007/s10008-013-2346-2.
16. Pocrifka, L.; Gonçalves, C.; Grossi, P.; Colpa, P.; Pereira, E. Development of RuO_2–TiO_2 (70–30)mol% for pH measurements. *Sens. Actuators B Chem.* **2006**, *113*, 1012–1016, doi:10.1016/j.snb.2005.03.087.
17. McMurray, H.; Douglas, P.; Abbot, D. Novel thick-film pH sensors based on ruthenium dioxide-glass composites. *Sens. Actuators B Chem.* **1995**, *28*, 9–15, doi:10.1016/0925-4005(94)01536-q.
18. Lonsdale, W.; Wajrak, M.; Alameh, K. Effect of conditioning protocol, redox species and material thickness on the pH sensitivity and hysteresis of sputtered RuO_2 electrodes. *Sens. Actuators B Chem.* **2017**, *252*, 251–256, doi:10.1016/j.snb.2017.05.171.

19. Lonsdale, W.; Maurya, D.; Wajrak, M.; Alameh, K. Effect of ordered mesoporous carbon contact layer on the sensing performance of sputtered RuO_2 thin film pH sensor. *Talanta* **2017**, *164*, 52–56, doi:10.1016/j.talanta.2016.11.020.
20. Liao, Y.-H.; Chou, J.-C. Preparation and characteristics of ruthenium dioxide for pH array sensors with real-time measurement system. *Sens. Actuators B Chem.* **2008**, *128*, 603–612, doi:10.1016/j.snb.2007.07.023.
21. Yao, X.; Vepsäläinen, M.; Isa, F.; Martin, P.; Munroe, P.; Bendavid, A. Advanced RuO_2 Thin Films for pH Sensing Application. *Sensors* **2020**, *20*, 6432, doi:10.3390/s20226432.
22. Chou, J.-C.; Liu, S.-I.; Chen, S.-H. Sensing Characteristics of Ruthenium Films Fabricated by Radio Frequency Sputtering. *Jpn. J. Appl. Phys.* **2005**, *44*, 1403–1408, doi:10.1143/jjap.44.1403.
23. Sardarinejad, A.; Maurya, D.K.; Alameh, K. The pH Sensing Properties of RF Sputtered RuO_2 Thin-Film Prepared Using Different Ar/O_2 Flow Ratio. *Materials* **2015**, *8*, 3352–3363, doi:10.3390/ma8063352.
24. Lonsdale, W.; Wajrak, M.; Alameh, K. Manufacture and application of RuO_2 solid-state metal-oxide pH sensor to common beverages. *Talanta* **2018**, *180*, 277–281, doi:10.1016/j.talanta.2017.12.070.
25. Xu, B.; Zhang, W.-D. Modification of vertically aligned carbon nanotubes with RuO_2 for a solid-state pH sensor. *Electrochim. Acta* **2010**, *55*, 2859–2864, doi:10.1016/j.electacta.2009.12.099.
